# Laparo-endoscopic combination for the safe extraction of an open safety pin in a 9-month-old child. Case report

**DOI:** 10.1016/j.amsu.2021.102716

**Published:** 2021-08-13

**Authors:** Silvia Bisoffi, Francesco Fascetti Leon, Elisa Zambaiti, Alberto Sgrò, Luca Maria Antoniello, Piergiorgio Gamba

**Affiliations:** Pediatric Surgery Unit, Women's and Children's Health Department, University of Padua, Padova, Italy

**Keywords:** Sharp foreign body, Open safety pin, Mini-invasive extraction, Child, Case report

## Abstract

**Introduction:**

and importance: Accidental ingestion of foreign bodies (FBs) is common among infants. In case of sharp FBs, the risk of accidental organ damage with potential life-threatening complications constitutes an absolute indication for removal. We present the case of a child, who, following the ingestion of an open safety pin, was successfully treated exclusively with minimally invasive techniques.

**Case presentation:**

A 9-month-old male patient was admitted for hematemesis. An anteroposterior and lateral X-ray of the thorax and abdomen revealed the presence of an open safety pin in the epi-mesogastric region, without a precise localization. Upper and lower gastrointestinal endoscopy, fluoroscopy, and laparoscopy were combined in the same intervention to localize and safely remove the foreign body. The patient was dismissed on a postoperative day 1.

**Clinical discussion and conclusion:**

The two main pitfalls of this scenario were the initially uncertain location of the foreign body and the young age of the patient. A combination of different techniques was used to safely locate and remove the foreign body, reducing hospitalization and avoiding repeated radiological exposure. An experienced team in a tertiary paediatric surgical and endoscopic centre increases the chances of success and minimizes invasiveness and the risk of complications.

## Highlights

• We report on a completely minimally invasive extraction of a sharp foreign body
• Hints to ensure optimal combination of laparoscopy and endoscopy are provided
• Importance of referral to a tertiary centre with expertise in paediatric endoscopy and minimally invasive surgery

## 1 Introduction

Accidental ingestion of foreign bodies (FBs) is a common event in the paediatric population. Up to 75% of all FBs ingestions occur in children around 5 years of age or younger [1] and about 20% of children aged 1–3 years have ingested a foreign body during their life [2]. As most FBs ingestions (over 97%) occur at home, the child is usually brought to the Emergency Departments (ED) by the parents that either assisted or discovered the ingestion [3]. The FBs most frequently ingested by children are coins, accounting for up to two thirds of the total evaluated cases in United States ED [3]. Toys, jewellery, and batteries follow with a cumulative ingestion rate of 25% (10% 7% and 7% respectively) [3].

The accidental ingestion of sharp foreign bodies (SFBs) constitutes an absolute indication for the removal, due to the high risk of damage of the gastrointestinal (GI) tract and surrounding parenchymatous organs. Delayed presentation and management increase the risk of complications [4]. In the literature, many major complications following SFBs ingestion exist, among which perforation of the heart by a swallowed safety-pin [5], abscess or fistula formation [6] and migration to the pancreas of an ingested needle [7] are the most peculiar. As from preoperative imaging, usually x-ray, it may often be hard to exactly locate the FB, the best surgical approach may be difficult to establish. The specialists involved in the primary assessment and treatment of FBs ingestion must thus be confident in the management of a variety of objects as the different types of FBs, as well as the location within the digestive system, may require peculiar approaches. We present the case of a child, who, following the ingestion of an open safety pin, underwent successful extraction using exclusively a combination of minimally invasive techniques. To our knowledge, this is the first report of a completely minimally invasive extraction of a sharp foreign body, and it may add a valid therapeutic option to the literature.

### 1.1 Presentation of case

**Fig. 1:**
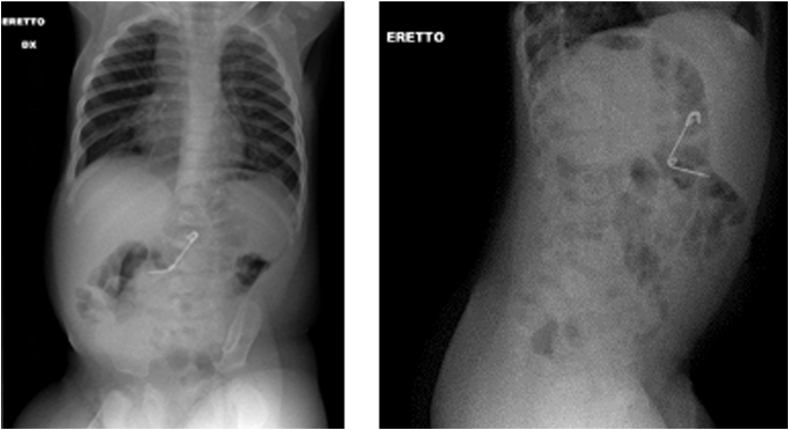
Anteroposterior (on the right) and lateral (on the left) X-ray of the thorax and abdomen.

A 9-month-old male patient was admitted to the ED of our tertiary paediatric centre, after an episode of hematemesis. Following the vomit, the mother noticed that a safety pin was missing from the bed where the patient slept. Past medical history of the patient and his family was uneventful. The patient was evaluated by the paediatric surgeon on call. The vital parameters were normal, and the patient presented in good clinical condition without any major symptoms, afebrile even if minimally irritable. During the clinical examination, all signs of alarm for a FB impacted in the upper gastrointestinal tract and respiratory regions, including drooling, vomiting, coughing, or stridor were excluded. An anteroposterior and lateral X-ray of the thorax and abdomen was performed, and an open safety pin was revealed in the epi-mesogastric region, without any evidence of bowel obstruction or pneumoperitoneum (Fig. 1). Nonetheless, the radiologic exam was unable to determine the exact localization of the pin.

**Fig. 2:**
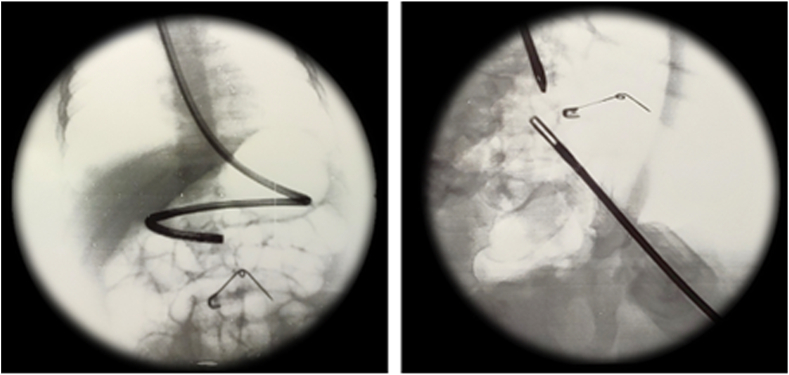
The triangulation with laparoscopic instruments and gastroscope under fluoroscopy allowed to localize the pin at the splenic flexure.

**Fig. 3:**
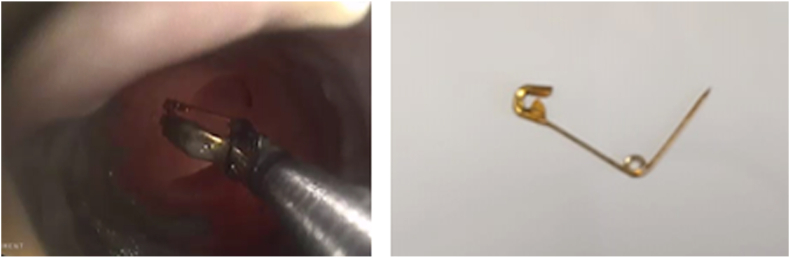
Endoscopic removal (on the left) and the open safety pin (on the right).

Considering the dimension, sharpness, and consequent damaging potential of the FB, the patient was sent for emergent esophagogastroduodenoscopy. Unfortunately, we could not document either the presence of the safety pin or any lesion in the oesophagus, stomach, and duodenum. Therefore, we proceeded with a diagnostic laparoscopy. An experienced team (two senior paediatric surgeons and one junior paediatric surgeon) performed the intervention. The intestine was carefully “palpated” with atraumatic laparoscopic forceps from the Treitz ligament to the ileocecal valve. No damages nor the site of the FB were still identified. Hence, under fluoroscopy the laparoscopic instruments were used to point and finally localize the pin at the splenic flexure (Fig. 2). Colonoscopy was thus performed; the atraumatic laparoscopic forceps aided to avoid insufflation of the whole colon by clamping the upstream segment. This artifice guaranteed an optimal laparoscopic view for a safe extraction (Fig. 3).

After the operation, the patient restarted a normal enteral feeding on postoperative day 1 with good tolerance. The postoperative pain was controlled with paracetamol only on the first postoperative day and the child remained afebrile during the entire hospital stay. He did not present any vomit. He was dismissed on postoperative day 1 and at the clinical follow-up he was completely asymptomatic, without any signs of surgical wounds infection or complication.

The parents showed high satisfaction with the resolution of a potentially life-threatening situation through exclusively minimally invasive techniques. As the initial expected clinical outcome was unknown because the exact location of the foreign body could not be defined preoperatively, the attained clinical outcome was especially favourable with an only one-night hospital stay.

This case report has been reported in line with the SCARE 2020 Criteria [8]. Consent was obtained from the parents of the child for publication of this case report and accompanying anonymized images.

## 2 Discussion

Foreign body ingestion occurs frequently in the paediatric population, especially between 6 months and 3 years [9]. The transit through the gastrointestinal tract is completed spontaneously in an average of 3.6 days [4]. Nevertheless, FB can impact at some point, causing dangerous consequences: about 20% of cases impact within the oesophagus where the FB can cause the formation of bronchoesophageal fistula, aortoesophageal fistula, oesophageal perforation with subsequent mediastinitis or abscess, complete oesophageal stricture or oesophageal obstruction, pulmonary oedema, oesophageal diverticulum [10,11]. The probability of impaction depends on the quality, shape, size of the foreign body, and the child's medical status. The initial evaluation usually consists of a clinical examination with a plain radiograph to directly localize a radiolucent FB or by contrast studies to determine indirect signs of a radiotransparent FB. When FBs are localized in abdomen, in most cases the X-ray images can reveal the type of the ingested foreign body [12], but it is often hard to exactly locate it. This may complicate the choice of the appropriate surgical approach and it might constitute a challenge for the surgeon trying to remove it.

The multiple possibilities occurring in the management of a child, who had ingested a foreign body, require complex skills for the paediatric surgeon, including the decision between intervention or not, experience in endoscopic retrieval, and in dealing with potentially life-threatening complications. The requirement of surgery or endoscopy, due to impaction, has not been quantified so far but it is estimated that about 20% of ingested foreign bodies may require endoscopic retrieval and in 1% of cases even surgical intervention [11]. Considering safety pins, it has been reported that 41% of safety pins transit spontaneously, 28.5% need an endoscopy, and 30.5% require surgical intervention [13], with the latter being more frequent in the case of open pins.

According to the American Society for Gastrointestinal Endoscopy Guidelines, the presence of an SFB represents an absolute indication of removal [14]. On the other hand, in 2015 the North American Society for Paediatric Gastroenterology, Hepatology, and Nutrition recommended conservative management for sharp foreign bodies, whose position is guaranteed to be below the stomach, in case of an asymptomatic patient [15]. Considering our specific case, the patient presented with hematemesis; moreover, it was impossible to identify the exact position of the foreign body and thus to determine whether it had passed the stomach or not. With the actual understanding of the real location of the SFBs at the splenic flexure, we might now speculate that hematemesis was either due to minimal lesions in the upper GI that were already healed at the time of endoscopy, or it may have been an incidental finding not related at all with the ingestion. However, the uncertainty of the location, together with the knowledge of the cases reported in the literature of damages along the GI tract beyond the pylorus, warranted intervention against observation. Besides, this latter approach would have obligated strict monitoring of the child for an estimated time of 4 days [4] and the repetition of X-rays to track the transit of the pin, while the operative approach allowed us to dismiss the patient on postoperative day 1. The objective of this study is therefore to report the eventuality that a sharp foreign body may not be exactly located at beginning and to provide a safe solution for its extraction with the use of exclusively minimally invasive techniques, obtaining an optimal result in terms of recovery, postoperative length of hospital stay, and parent satisfaction.

We had some advantages from the reported combined technique: the use of radiograms and laparoscopic instruments allowed quick and precise identification of the position of the foreign body; second, laparoscopy provided an accurate control of any potential lesion during the endoscopic handling of the pin.

In the literature, there are only a few cases reported of colonoscopic removal of potentially dangerous foreign bodies in adults and only one case of a safety pin in a child extracted through colonoscopy performed after 4 days of hospitalization [11]. Success rates of the removal depend on the experience level of the endoscopist and device choice [15]. One doubt remains if the repetition of an X-ray immediately before the operation would have helped the localization of the ingested pin. Nevertheless, considering the multiple images needed in combination with laparoscopic devices to localize the FB, it is unlikely that one single radiogram would have been sufficient.

## 3 Conclusion

In conclusion, we present a safe and totally minimally invasive approach for the removal of a sharp foreign body. Different minimally invasive techniques were combined during the same intervention leading to safe extraction of the open safety pin, with the prompt discharge of the patient on the first postoperative day. The risk for FB ingestion with potentially life-threatening conditions remains constant among paediatric patients. Paediatric surgeons should individualize care by providing the best and minimally invasive option updated with currently available techniques. We can, therefore, conclude that such complex cases must be centralized in tertiary centres with expertise in paediatric endoscopy and minimally invasive surgery.

## Authorship

All authors attest that they meet the current ICMJE criteria for Authorship.

## Author contribution

SB was the surgeon on call and described the study. FFL and AS performed the laparoscopy. LMA performed the endoscopy. EZ was the physician of the patient. FFL and PG prompted the clinical choice. All authors took part in writing the manuscript, reviewed it and revised the intellectual and technical content of the paper.

## Provenance and peer review

Not commissioned, externally peer-reviewed.

## Sources of funding

This research did not receive any specific grant from funding agencies in the public, commercial, or not-for-profit sectors. Work was presented at 50th National Conference, 22–24th of October 2019, Palermo. The material has not been published or submitted for publication elsewhere.

## Ethical approval

A formal ethical approval is not required at our institution to report single cases with anonymized data and images, especially when standard of care treatment is provided (without technical innovation or innovative materials).

## Consent of patient

Written informed consent was obtained from the parents of the child for anonymized publication of this case report and accompanying images. A copy of the written consent is available for review by the Editor-in-Chief of this journal on request.

## Research registration

Not needed. We report a of combination of already established and known techniques and devices. No totally new techniques or devices have been used.

## Guarantor

S.B. as the guarantor accepts full responsibility for the work and the conduct of the study, had access to the data, and controlled the decision to publish.

## Declaration of competing interest

All authors have no financial disclosures, neither other conflict of interest.
